# Corrigendum to “Potential Extensions of the US FRAX Algorithm”

**DOI:** 10.1155/2018/3021801

**Published:** 2018-04-02

**Authors:** Journal of Osteoporosis

In the article titled “Potential Extensions of the US FRAX Algorithm” [1], the authors wish to correct the affiliation of John A. Kanis. This should have been listed as Centre for Metabolic Bone Diseases, University of Sheffield Medical School, Sheffield S10 2RX, UK. In the section on FRAX® calculations, “Fracture probabilities were calculated by the World Health Organization Collaborating Centre for Metabolic Bone Diseases” should read “Fracture probabilities were calculated by the Centre for Metabolic Bone Diseases.”

In the Introduction, FRAX is described as “the World Health Organization's fracture risk assessment tool”. The Metabolic Bone Diseases Unit at the University of Sheffield that developed FRAX was a WHO Collaborating Centre from 1991 to 2010 and FRAX is based on data generated from that centre. However, the WHO and the University of Sheffield say FRAX was not developed or endorsed by the WHO [2]; Professor Kanis disagrees with that position.

## References

[1] Joseph Melton III L., Atkinson E. J., Achenbach S. J. (2012). Potential Extensions of the US FRAX Algorithm. *Journal of Osteoporosis*.

[2] Ford N., Norris S. L., Hill S. R. (2016). Clarifying WHO’s position on the FRAX® tool for fracture prediction. *Bulletin of the World Health Organization*.

